# LncRNAs Predicted to Interfere With the Gene Regulation Activity of *miR-637* and *miR-196a-5p* in GBM

**DOI:** 10.3389/fonc.2020.00303

**Published:** 2020-03-09

**Authors:** Jingfang Zheng, Zhiying Su, Yang Kong, Qingping Lin, Hongli Liu, Yanlong Wang, Jian Wang

**Affiliations:** ^1^Department of Gynecology, Women and Children's Hospital, School of Medicine, Xiamen University, Xiamen, China; ^2^Department of Reproductive Medicine, Women and Children's Hospital, School of Medicine, Xiamen University, Xiamen, China; ^3^Department of Neurosurgery, Qilu Hospital of Shandong University and Institute of Brain and Brain-Inspired Science, Shandong University, Jinan, China; ^4^Translational Cancer Research Group, Department of Biomedicine, University of Bergen, Bergen, Norway

**Keywords:** lncRNA, mRNA, GBM, molecular datasets, network

## Abstract

Rigorous molecular characterization of biological systems has uncovered a variety of gene variations underlying normal and disease states and a remarkable complexity in the forms of RNA transcripts that exist. A recent concept, competitive endogenous RNA, suggests that some non-coding RNAs can bind to miRNAs to modulate their role in gene expression. Here, we used several platforms, integrating mRNA, non-coding RNAs and protein data to generate an RNA-protein network that may be dysregulated in human glioblastoma multiforme (GBM). Publicly available microarray data for mRNA and miRNA were used to identify differentially expressed miRNAs and mRNAs in GBM relative to non-neoplastic tissue samples. Target miRNAs were further selected based on their prognostic significance, and the intersection of their target gene set with the differentially expressed gene set in Venn diagrams. Two miRNAs, miR-637 and miR-196a-5p, were associated with poor and better prognosis, respectively, in GBM patients. Non-coding RNAs, ENSG00000203739/ENSG00000271646 and TPTEP1, were predicted to be miRNA target genes for miR-637 and miR-196a-5p and positively correlated with the selected mRNA, CYBRD1 and RUFY2. A local protein interaction network was constructed using these two mRNAs. Predictions based on the ENSG00000203739/ENSG00000271646-miR-637-CYBRD1 and TPTEP1-miR-196a-5p-RUFY2 regulation axes indicated that the two proteins may act as an oncogene and tumor suppressor, respectively, in the development of GBM. These results highlight competitive endogenous RNA networks as alternative molecular therapeutic targets in the treatment of the disease.

## Introduction

Glioblastoma multiforme (GBM) is the most aggressive central nervous system tumor in adults, and the prognosis is bleak. Conventional therapies, including surgery, radiotherapy, and chemotherapy with temozolomide have not resulted in significant improvement in the survival outcomes of patients with GBM. Median overall survival is 15–23 months, and 5-year survival is <6% (1). Causes of poor prognosis include invasive tumor growth in an essential organ that cannot be thoroughly removed, the presence of the blood-brain barrier (BBB), their intrinsic resistance to the induction of cell death, tumor heterogeneity and a complex pathogenesis. The basis for this behavior is the simultaneous corruption of many genes which results in the lack of a single, targetable oncogenic pathway, and thus, significant challenges in systemic therapy.

Answers for treatment are thought to be buried in the molecular datasets accumulating since 2006 when The Cancer Genome Atlas (TCGA) team, sponsored by the National Cancer Institute (NCI), published DNA copy number, gene expression, and DNA methylation analysis for 206 GBMs. Rigorous analysis of these data has led to some critical insights in the development of human gliomas. GBMs can now be classified as one of four molecular subtypes based on transcriptome expression data: classical, neural, mesenchymal, and proneural. Subsequent analysis of the methylation status of DNA promoter regions in 272 GBMs revealed two major glioma-CpG island methylation phenotypes (glioma-CpG island methylator phenotype, or G-CIMP and non-G-CIMP types). Finally, a total 1,122 gliomas samples were divided into *IDH* mutated and wild type tumors based on analysis of the multi-dimensional histological data.

Non-coding RNAs have also become part of the story. A collection of dysregulated lncRNAs, including hundreds of candidate onco- and tumor-suppressor lncRNAs, have been identified in the context of 14 different tumor types (2). Recurrent hypomethylation of 1,006 lncRNA genes in cancer, including *EPIC1* (epigenetically-induced lncRNA1) has also been described (3). *EPIC1* promotes cell-cycle progression by interacting with MYC, enhancing luminal B breast cancer cell growth *in vitro* and *in vivo*.

The expanding landscape for RNA transcript types has triggered additional theories about gene regulation. A recent concept, competitive endogenous RNA (ceRNA), represents a novel regulatory mechanism between non-coding and coding RNAs. The theory suggests that lncRNAs, cirRNAs, and pseudogenes can act as “molecular sponges” to compete for miRNAs and effectively modulate their functions. The competition for miRNAs is mediated by miRNA binding sites or miRNA response elements (4). This creative hypothesis is supported by an increasing number of experimental results.

With the development of high-throughput gene sequencing and chip technology, analyzing molecular data has become an extremely meaningful but challenging task. An increasing number of R language packages and bioinformatics analysis tools have become more user friendly for a broader range of investigators. Here, we used some of these tools to analyze miRNA and mRNA datasets to determine where they might converge in the development of human GBM. Our results led us to two miRNAs, *miR-196a-5p* and *miR-637*, their target mRNAs encoding a putative oncogene and tumor suppressor, and non-coding RNAs regulating the miRNA activity. We show how a fundamental biological question, which genes and miRNAs are differentially expressed in human GBM, can provide the basis for the construction of a molecular network including RNAs and proteins that might drive aspects of GBM development. Such “excavation” of molecular datasets is the key for the advancement of novel therapies in the treatment of the disease.

## Materials and Methods

### Microarray Data

MicroRNA expression profiles in GSE25631 from the publicly available NCBI GEO database, which had been collected using the Illumina GPL8179 platform (Human v2 microRNA Expression Beadchip), were analyzed. The GSE25631 dataset includes 82 primary GBM surgical specimens and 5 non-neoplastic brain tissue samples from areas surrounding arteriovenous malformations as controls. mRNA expression profiles were obtained from the GSE4290 dataset (5), which is based on the Affymetrix GPL570 platform (HG U133 Plus 2.0 Array). The GSE4290 dataset includes 79 GBM samples and 23 non-neoplastic brain tissue samples from epilepsy patients as controls. To validate our results, GSE90604 and GSE65626, two microRNA expression datasets, were also analyzed.

### Analysis to Identify Differentially Expressed microRNAs and Differentially Expressed Genes (DEGs)

Analysis using GEO2R, a webtool available from the NCBI, was performed to detect differentially expressed microRNAs and DEGs between GBM and non-neoplastic control samples. Details of the R script of GEO2R are provided in the Supplementary Material. Adjusted *P*-values were used to reduce the false positive rate using the Benjamini and Hochberg false discovery rate method by default (6, 7). *P* < 0.05 and |logFC| ≥ 2 were set as the cutoff values.

### Identification of Target Genes of Candidate microRNAs

Cytoscape, open-source software for the integration of molecular interaction network data, was used to visualize the relationship between microRNAs and differentially expressed genes (DEGs). CyTargetLinker (8), a plug-in for Cytoscape, was used to identify microRNA-target genes (MTGs), based on experimentally validated microRNA-target interaction (MTIs) files stored in miRTarBase (9), a database containing miRNA-target interactions. In general, the collected MTIs in miRTarBase have been validated experimentally using luciferase assays, western blots, microarrays and next-generation sequencing.

### GO and KEGG Pathway Enrichment Analysis for MTGs of Candidate microRNAs and DEGs

Kyoto Encyclopedia of Genes and Genomes (10) (KEGG) pathway analysis was performed to identify potential functions of the MTGs of the candidate microRNAs and DEGs. Gene ontology analysis (GO), a common useful method for annotating genes and identifying characteristic biological attributes, including biological processes, molecular functions, and cellular components, for high-throughput genome or transcriptome data (11), was performed on DEGs. Metascape (http://metascape.org), a web-based online bioinformatics resource that aims to provide tools for the functional interpretation of large lists of genes or proteins (12), was also used to identify function of MTGs and to conduct GO and KEGG pathway enrichment (13) on DEGs derived in our analysis. The enriched KEGG pathways of MTGs were visualized using ClueGO+Cluepedia, a plug-in that visualizes the non-redundant biological terms for large clusters of genes in a functionally grouped network (14). For DEGs, visualization of the biological processes, molecular functions, cellular components and pathways was performed using Excel and R ggplot2 packages.

### Identification of Hub Genes Among DEGs

Protein names encoded by DEGs were imported into STRING (https://string-db.org/) to obtain a protein-protein interaction (PPI) network (15). CentiScaPe 2.2 was used to analyze nodes in the network (16). Genes with the highest degrees of connectivity were selected as hub genes. Analysis of the core genes can represent whether the chip results are consistent with GBM.

### Identification of Candidate Genes Regulated by DEGs and MTGs

Venn diagrams (17) were used to identify the intersection between *miR-196a-5p* target and GBM down-regulated genes, as well as between *miR-637* target and GBM up-regulated genes. Gene Expression Profiling Interactive Analysis (GEPIA; http://gepia.cancer-pku.cn/index.html), a newly developed interactive web server, was used to analyze differences in expression between tumor and normal samples using RNA sequencing data (18). A boxplot was generated to visualize the relationship.

### Identification of Target Non-coding RNAs of Candidate microRNAs

Analysis using LncBase v.2 was performed to predict the target non-coding RNAs of differentially expressed microRNAs in GBM (19). To acquire high confidence target non-coding RNAs, the threshold was set at > 0.9, and the tissue was confined to brain. Target non-coding RNAs of candidate genes, including lncRNAs, cirRNAs, and pseudogenes, were chosen based on a positive relationship with candidate genes in the data collected from TCGA GBMs on the Tanric website (20). The expression of target non-coding RNAs and candidate genes was set to a positive correlation above moderate levels (correlation coefficient > 0.4, *P*-value <0.01). The intersection between candidate genes and predicted target non-coding RNAs was made.

### PPI Network Extension and Establishment of the Competitive Endogenous RNA (ceRNA) Hypothesis

Protein names encoded by candidate genes were imported into STRING, a database of known and predicted protein-protein interactions (https://string-db.org/) (21). PPI networks were extended until the proteins from our analysis connected with each other. Candidate microRNA and non-coding RNAs were then mapped to the network.

## Results

### Identification of Differentially Regulated Candidate miRNAs in GBM

**Figure 1:**
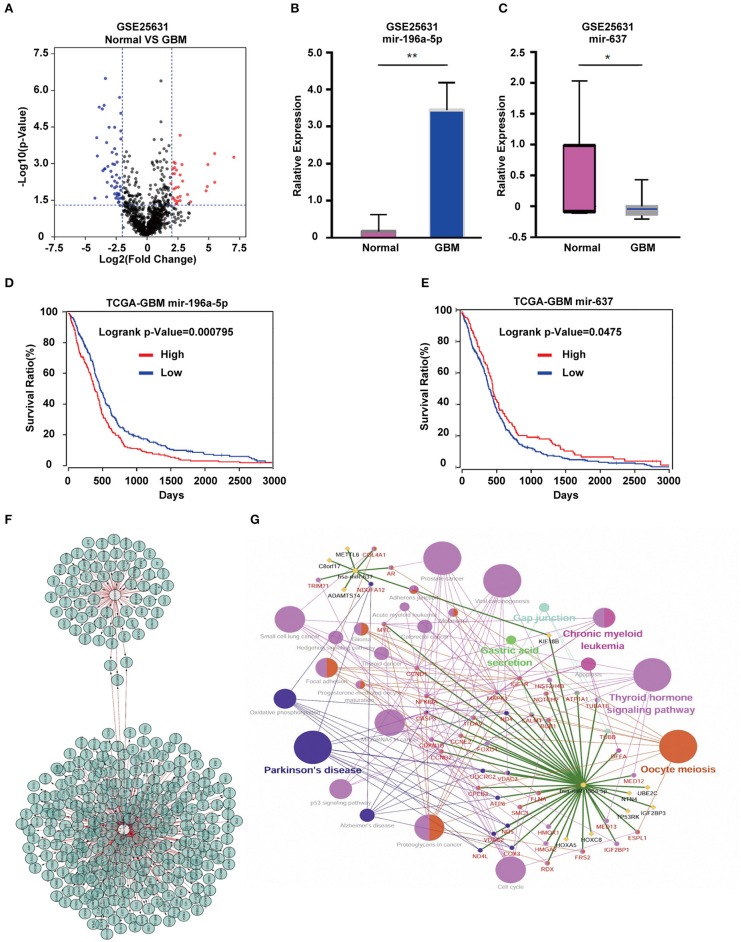
Identification of candidate miRNAs and prediction of their target genes. **(A)** Volcano plot compiled using expression data obtained from the publicly available dataset GSE25631. Red and blue dots represent up-regulated and down-regulated differentially expressed microRNAs, respectively (*P* < 0.05, |logFC| ≥ 2). **(B)** The expression of *miR-196a-5p* and **(C)** *miR-637* in GBM relative to non-neoplastic brain tissue samples. **(D)** Prognostic value of *miR-196a-5p* **(E)** and *miR-637* in GBM based on the TCGA database. **(F)** Predicted target genes of *miR-196-5p* and *miR-637*. **(G)** KEGG pathway enrichment analysis of target genes of *miR-196-5p* and *miR-637*. Figures in **(F,G)** were designed using the open source software Cytoscape 3.6.1 and its plugin or app CyTargetLinker, ClueG+Cluepedia. Data are shown as the mean ± standard deviation. ^*^P < 0.05; ^**^P < 0.01 vs. control samples.

Analysis of the GSE25631 dataset yielded a total of 67 differentially expressed miRNAs (*P* < 0.05 and |logFC| ≥ 2) between GBM and non-neoplastic brain. Of these miRNAs, 27 were up-regulated and 40 were down-regulated in GBM relative to control samples (Figure 1A). We examined the prognostic value of 10 miRNAs with the most significant fold changes in expression (Table 1), using OncoLnc, a tool for interactively exploring survival correlations coupled to expression data for mRNAs, miRNAs, or lncRNAs. Two of these miRNAs, *miR-196a-5p* and *miR-637*, were associated with overall survival (OS; Figures 1B,C). High expression of *miR-196a-5p* in GBMs (HR = 0.196, *P* = 0.000795) was associated with worse OS in patients (Figure 1D), while high expression of *miR-637* (HR = −0.634, *P* = 0.045) was associated with better OS in patients (Figure 1E). Because the statistical difference in OS using *miR-637* was not significant, we selected multiple cutoff values for verification. The results are shown in Supplementary Figure 1. The expression of *miR-196-5p* and *miR-637* was also verified in the TCGA database. The expression of *miR-196-5p* was consistent with the results obtained with the GSE25631 dataset. However, compared with the control group, *miR-637* was not significantly reduced in GBM from the TCGA (Supplementary Figures 2A,B).

**Table 1:** Top 10 differentially expressed miRNAs in GSE25631.

**miRNA_ID**	***P*-value**	**Log_**2**_FC**
hsa-miR-196a-5p	4.38E-04	5.599188
hsa-miR-558	6.37E-03	5.553814
hsa-miR-144	1.19E-03	5.045044
hsa-miR-106a	9.89E-03	4.979875
hsa-miR-637	2.93E-02	−4.14014
hsa-miR-876-3p	9.70E-05	−3.97221
hsa-miR-1224-5p	5.50E-06	−3.78824
hsa-miR-518e	4.09E-02	3.611791
hsa-miR-138-2-3p	6.31E-06	−3.54098
hsa-miR-203	2.13E-03	−3.45736

### KEGG Enrichment Analysis Links *miR-196-5p* and *miR-637* to Pathways Involved in Cancer

To understand the possible function of *miR-196-5p* and *miR-637* in the development of GBM, KEGG pathway enrichment analysis of their target genes was performed. KEGG analysis was performed on the *miR-196-5p* and *miR-637* target genes (*n* = 356) identified using Cytoscape (Figure 1F). The results revealed these genes to be associated with several pathways involved in disease development, including small cell lung cancer, proteoglycans in cancer, Parkinson's disease, viral carcinogenesis, prostate cancer, chronic myeloid leukemia, the Hedgehog signaling pathway, glioma, microRNAs in cancer, and the cell cycle (Figure 1G). We further validated the results using the open-source pathway database REACTOME (https://reactome.org/) (Supplementary Figures 3A,B) (22).

### Identification of Differentially Expressed Genes (DEGs) and Enrichment Analysis

**Figure 2:**
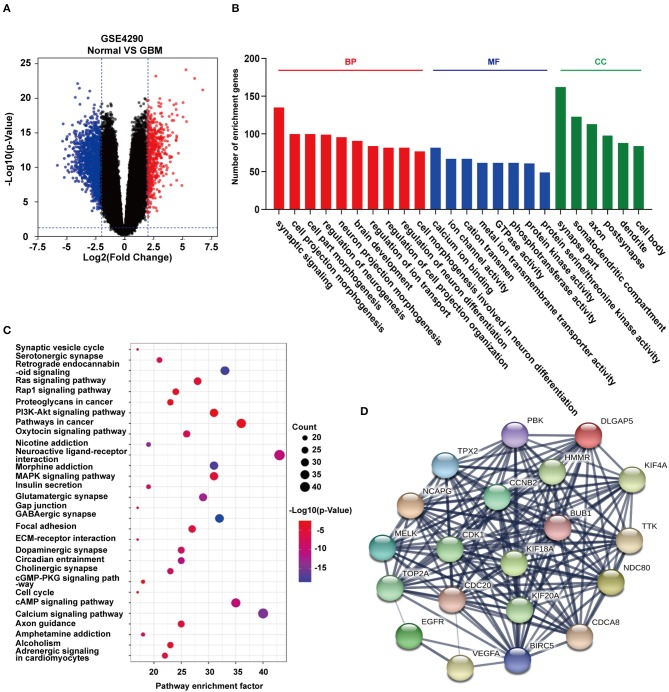
GO function and KEGG pathway enrichment analysis of DEGs and hub genes in GSE4290. **(A)** Volcano plot derived using the data in GSE4290. Red and blue dots represent up-regulated and down-regulated DEGs, respectively (*P* < 0.05 plus |logFC| ≥ 2). **(B)** Statistically enriched biological processes, molecular functions, and cellular components identified using GO function analysis of the DEGs. **(C)** Top 30 enriched pathways identified using KEGG pathway analysis of DEGs in GSE4290. **(D)** Top 20 hub genes predicted from the DEGs. These hub genes were representative genes involved in occurrence and progression of GBM. The figure in **(D)** was designed using the open source software Cytoscape3.6.1 and its plugin or the app STRING.

To identify differentially expressed genes in GBM relative to non-neoplastic brain, we performed analysis on the GSE4290 dataset containing mRNA expression profiles (5). A total of 1,170 differentially expressed genes were detected; 397 were up-regulated and 773 were down-regulated in GBM samples relative to non-neoplastic brain tissue (Figure 2A). To associate function with the DEGs, we performed GO (Figure 2B) and KEGG pathway analysis (Figure 2C). DEGs were found to be enriched in biological processes (BP, Figure 2B) involving the regulation of neurogenesis, plasma membrane bounded cell projection morphogenesis, cell morphogenesis involved in neuron differentiation, neurotransmitter secretion, and regulation of neurogenesis. They were also enriched in molecular functions (MF, Figure 2B) involving ion channel activity, protein kinase activity, and cell adhesion molecule binding. Based on cellular components (CC, Figure 2B), DEGs were found to be located in the synapse, the vesicle membrane, the perinuclear region of the cytoplasm, and the extracellular matrix. KEGG pathway enrichment analysis linked DEGs to processes involving the synaptic vesicle cycle, pathways in cancer, PI3K-Akt signaling, proteoglycans in cancer, and Ras signaling. These results suggest that these DEGs may play a role in promoting tumor progression through their function.

### Analysis of DEGs Interaction Network and Acquisition of Hub Genes

Using the STRING protein databases, we generated a PPI network for the top 20 hub genes with the highest degrees of connectivity (Figure 2D). The top 20 included genes known to promote the development of human cancer. *CDK1, CCNB2*, and *CDC20* (23) are involved in the regulation of the cell cycle. *EGFR* and *VEGFA* have been reported to promote GBM proliferation and invasion. *TOP2A, BUB1, NDC80*, and *TTK* participate in mitosis. *BIRC5* is a member of the inhibitor of apoptosis (IAP) gene family, which prevents apoptotic cell death. Finally, *HMMR* encodes a protein involved in cell motility.

### Identification of Candidate Genes Regulated by *miR-196-5p*/*miR-637*

**Figure 3:**
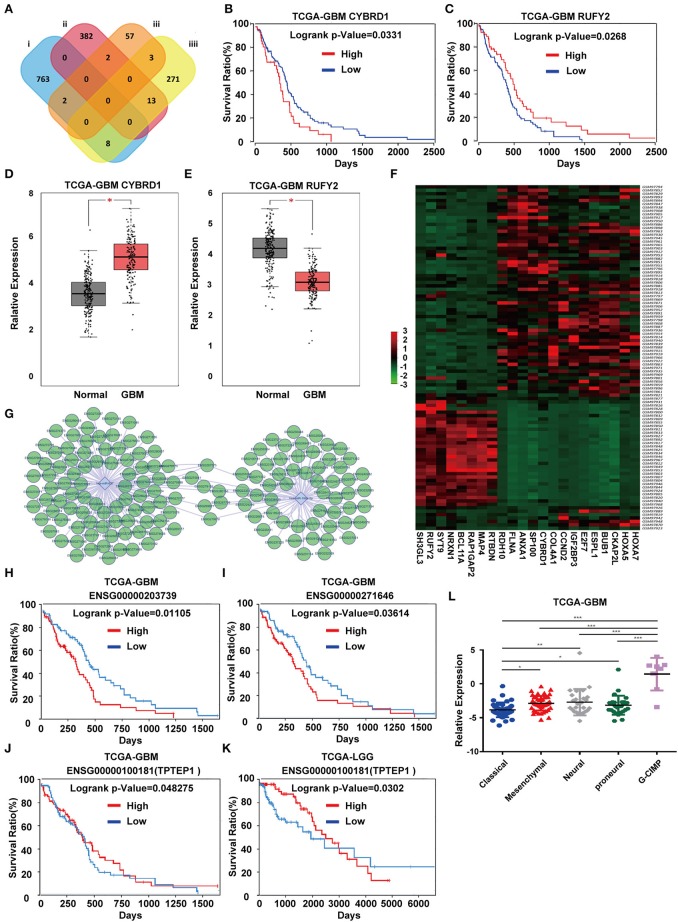
Identification of candidate genes and construction of a ceRNA network. **(A)** Venn diagrams showing the intersection between predicted target genes of *miR-196-5p*/*miR-637* and DEGs. Kaplan-Meier plots showing the prognostic value of **(B)** *CYBRD1* and **(C)** *RUFY2* in GBM based on the TCGA database. The expression of **(D)** *CYBRD1* and **(E)**. *RUFY2* in GBM relative to non-neoplastic brain tissue samples in the TCGA database. **(F)** Heatmap displaying differential expression of intersecting genes between the GBM and control groups in GSE4290. **(G)** Target non-coding RNAs of *miR-196-5p* and *miR-637*. Kaplan-Meier plots showing the prognostic value of **(H)** *ENSG00000203739* and **(I)** *ENSG00000271646* in GBM, and *ENSG00000100181* (*TPTEP1*) in **(J)** GBM and **(K)** LGG based on the TCGA database. **(L)** Expression of *TPTEP1* in GBM molecular subtypes based on the TCGA database. The figure in **(A)** was designed using the open source software Cytoscape3.6.1. Data are shown as the mean ± standard deviation. ^*^P < 0.05; ^**^P < 0.01; ^***^P < 0.001 vs. control samples.

To identify DEGs that may be regulated by *miR-196-5p*/*miR-637*, we generated a venn diagram illustrating the intersection between the target genes of *miR-196-5p*/*miR-637* and the DEGs (Figure 3A). Among the genes appearing in the intersection of the two datasets were *CYBRD1* and *RUFY2* (Table 2). Expression levels were high and low for *CYBRD1* and *RUFY2*, respectively, in GBM, corresponding with activity as a putative oncogene or tumor suppressor gene. The expression of these two genes was also related to the OS of patients (Figures 3B,C). High expression of *CYBRD1* was related to poor survival whereas high expression of *RUFY2* was associated with better survival. The differential mRNA expression of *CYBRD1* and *RUFY2* was consistent with results obtained using the expression data from the TCGA GBMs (Figures 3D,E). We also analyzed the relationship between the expression of *CYBRD1*/*RUFY2* and *MGMT*/*IDH* status within TCGA GBM samples. Neither mRNA exhibited significant differences between methylated/unmethylated *MGMT* and wild-type/mutant *IDH* in tumors (Supplementary Figure 4). Finally, a heatmap based on mRNA expression data of intersection genes from GSE4290 illustrates the differential expression of the two genes between GBM and non-neoplastic samples (Figure 3F).

**Table 2:** Intersection of non-coding RNAs involved in the regulation of miR-196-5p/miR-637 and CYBRD1/RUFY2.

**MicroRNA**	**Prediction score**	**Non-coding RNA**	**Gene**	**Correlation coefficient**	***P*-value**
hsa-miR-637	1	ENSG00000203739	CYBRD1	0.401	3.17E-07
hsa-miR-637	0.999	ENSG00000246263	CYBRD1	0.425	5.35E-08
hsa-miR-637	0.999	ENSG00000254154	CYBRD1	0.417	9.96E-08
hsa-miR-637	0.999	ENSG00000271646	CYBRD1	0.445	1.09E-08
hsa-miR-637	0.998	ENSG00000272908	CYBRD1	0.403	2.89E-07
has-196a-5p	0.904	ENSG00000100181	RUFY2	0.421	2.78E-05

### Identification of Non-coding RNAs Involved in the Regulation of *miR-196-5p*/*miR-637* and *CYBRD1*/*RUFY2*

To determine whether any non-coding RNAs might be involved in the regulation of *CYBRD1* and *RUFY2*, we generated a diagram to reveal the intersection between predicted target ncRNAs of *miR-196a-5p*/*miR-637* and non-coding RNAs which are positively related to these two genes (Figure 3G). Three ncRNAs, *ENSG00000203739, ENSG00000271646*, and *ENSG00000100181*, emerged from the analysis, and high expression of *ENSG00000203739* and *ENSG00000271646* was associated with poor prognosis in GBM using Log-Rank models (Figures 3H,I). The survival curve for the third ncRNA, *ENSG00000100181*, was only statistically significant in the Cox model for GBM and low grade glioma (Figures 3J,K).

The official symbol of *ENSG00000100181* is *TPTEP1*, which is also known as *psiTPTE22*. psiTPTE22-HERV has been reported to be epigenetically silenced by DNA methylation in cancers of the kidney, liver, lung, and stomach (24–26). We were therefore interested in the possibility that *TPTEP1* expression might differ on the basis of GBM molecular subtype. Using the TCGA database, we found expression of *TPTEP1* to be significantly higher in the G-CIMP subtype relative to the other molecular subtypes. As patients with G-CIMP subtype tumors in general have a better prognosis, low-expression of *TPTEP1* in non-G-CIMP subtypes may be consistent with a role as a tumor suppressor gene (Figure 3L). Therefore, we believe the role of *TPTEP1* in the pathogenesis of GBM warrants further investigation.

### A Competitive Endogenous RNA (ceRNA) Regulation Network Involving *TPTEP1*, CYBRD1, and RUFY2 Built in GBM

**Figure 4:**
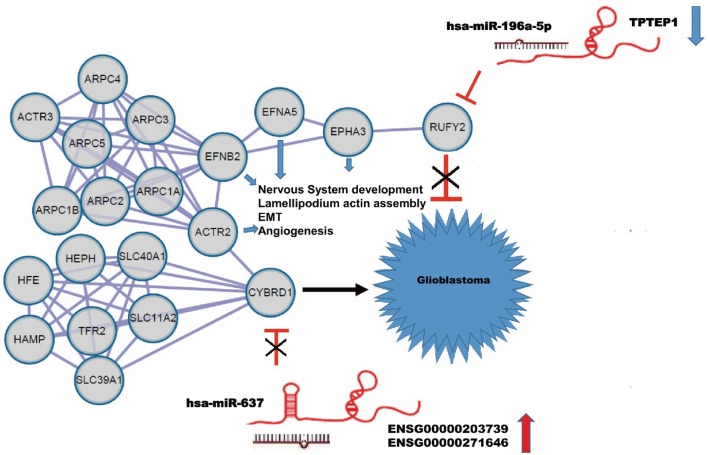
Construction of a ceRNA regulation network in GBM based on differentially expressed miRNAs and DEGs. CeRNA network where gray circles represent predicted proteins. High-expression of *ENSG00000203739*/*ENSG00000271646* promotes GBM proliferation and invasion by suppressing *miR-637* and promoting expression of the putative oncogene *CYBRD1*. Down-regulation of *TPTEP1* fails to adsorb/bind to *miR-196a-5p*. The overexpression of *miR-196a-5p* impedes translation of the putative tumor suppressor RUFY2. Dysregulation at both points in the network potentially contributes to progression of GBM. EFNA5, FNB2, ACTR2, EPHA3 also act as putative oncogenes.

Using the String database, we constructed a local protein network between the proteins CYBRD1 and RUFY2 (Figure 4). We then integrated the ncRNAs. In this network, high-expression of *ENSG00000203739*/*ENSG00000271646* was predicted to promote GBM proliferation and invasion by suppressing *miR-637* which leads to increased expression of CYBRD1, a putative oncogene. Loss of *TPTEP1* however leads to increased levels of *miR-196a-5p*/to adsorpt/bind to *miR-196a-5p*. When overexpressed, *miR-196a-5p* impedes translation of RUFY2, a putative tumor suppressor protein. Thus, dysregulation of these ncRNAs can lead to progression of GBM through gain and loss of the CYBRD1 and RUFY2, respectively.

The local PPI network also included the proteins EFNA5, EFNB2, ACTR2, EPHA3. Many of these proteins are implicated in GBM development. EFNA5, for example, is a member of the ephrin gene family, which participates in late stage nervous system development and differentiation. EFNB2 plays an important role in physiological and pathological angiogenesis, and its role in tumor vessel development has been extensively studied. EFNB2 has been shown to mediate perivascular invasion of glioblastoma stem-like cells (27). ACTR2 is known to be a major constituent of the ARP2/3 complex, which is located at the cell surface and is essential for cell shape and motility through lamellipodial actin assembly and protrusion. Overexpression of ARP2 has been shown to promote gastric cancer cell migration and invasion. In contrast, ARP2 knockdown suppressed cell motility (28). Finally, *EPHA3* is frequently overexpressed in GBM and in particular, in the mesenchymal molecular subtype, which also shows a more aggressive phenotype in patients (29). Importantly, *EPHA3* is highly expressed on the tumor-initiating cell population in glioma, which potentially maintains tumor cells in a less differentiated state by modulating mitogen-activated protein kinase signaling.

The results, therefore, could be consistent with a regulatory network composed of ceRNA plus the interactive proteins CYBRD1 and RUFY2 and involved in the proliferation and invasion of GBM.

## Discussion

The challenge today is in analyzing burgeoning molecular datasets in ways that will yield biologically and clinically meaningful insights into the development and treatment of human disease. In the current study, we explored the integration of RNA and protein datasets to identify pathways regulating the development of human GBM. We first analyzed standard array data to identify differentially expressed microRNAs and mRNAs in GBM relative to non-neoplastic brain tissue controls using the data contained in the StarBase, LncBase, Tanric, and TCGA databases. Based on the theory of ceRNA, we found potential ncRNA regulatory pathways involving an oncogene and a tumor suppressor, *ENSG00000203739*/*ENSG00000271646*-*miR-637*-CYBRD1 and *TPTEP1*-*miR-196a-5p*-RUFY2, and constructed a local PPI network which might contribute to proliferation and invasion of GBM.

Experimental results are consistent with some of our predictions. First, Boult et al. have reported that CYBRD1 was overexpressed in the progression of Barrett's metaplasia to adenocarcinoma and this change are associated with increased iron deposition (30). Brookes et al. also confirmed that the increased expression in iron import proteins, including CYBRD1, was associated with progression to colorectal cancer (31). However, other studies showed that high CYBRD1 expression has been associated with increased metastasis- and/or relapse-free survival in breast cancer (32). In functional experiments, CYBRD1 inhibited phosphorylation of FAK and the focal adhesion pathway which is involved in migration and invasion. The reasons for these discrepancies include differences in pathogenesis and the shift between proliferation and migration in tumors. Second, *miR-196a* has been reported to function as a putative oncogene in many cancers. *MiR-196a* is significantly upregulated in GBM, and high levels of *miR-196a* are positively related to the malignant progression of gliomas (33). Third, *miR-196a-5p* promotes proliferation and suppresses apoptosis in GBM cells both *in vitro* and *in vivo* by targeting IkBa (34). *MiR-196a-5p* also interacts directly with the 3'UTR of *ZMYND11* and promotes the growth of GBM cells (35). Finally, *miR-637* has been reported to be a tumor suppressor gene in diverse human cancers, such as gastric (36), ovarian (37) and colorectal cancers (38). Results in human glioma are consistent with a function as a tumor suppressor; expression levels of *miR-637* were significantly reduced in clinical glioma tissues compared with normal brain tissues (39). Moreover, these studies revealed that *miR-637* directly binds AKT1 and inhibits glioma cell growth, migration and invasion *in vitro* and *in vivo*. These data support the feasibility of our approach.

There are however deficiencies in our strategy. First, excluding function as a “molecular sponge,” the mode of action of lncRNAs can be roughly divided into the following categories: signal, guide and scaffold. Second, due to the complexity of molecular mechanisms regulating disease development and the limitations of our analytical methods, many important molecules involved in GBM remain unidentified. Third, we input more processed TCGA data into our analysis, which may result in the loss of a more comprehensive perspective. Finally, because many algorithms are based on computer models, many prediction results cannot be achieved in real-life experiments.

Significant effort to analyze and integrate such large amounts of molecular data are taking place worldwide. Researchers funded by the National Institutes of Health (NIH), for example, have completed a comprehensive genomic analysis known as the PanCancer Atlas. This project published a total of 27 top bioinformatics papers. These articles are undoubtedly innovative and encouraging. But such efforts are merely the beginning. Here, we laid the groundwork for a new strategy to explore the complicated molecular mechanisms underlying the development of GBM or other diseases. The next important step is to verify our approach using functional experiments to confirm our model in Figure 4.

## Data Availability Statement

The datasets generated for this study can be found in the GSE25631, GSE4290, GSE90604, GSE65626.

## Author Contributions

JZ, YK, and JW contributed to the conception of the study. JZ and YK contributed to experimental technology and experimental design. JZ, ZS, YK, QL, and HL performed the data analyses. JZ, YK, and JW wrote the manuscript. YW and JW supervised the study.

### Conflict of Interest

The authors declare that the research was conducted in the absence of any commercial or financial relationships that could be construed as a potential conflict of interest.

## Supplementary Material

Supplementary Figure 1 Survival curves for the upper 20% of patients with high hsa-miR-637 expression in comparison with the lower 20%, 30%, 40%, 50%, 60%, 70%, and 80% of patients with low hsa-miR-637 expression **(A–G)**. Survival curve for the upper 30% of patients with high hsa-miR-637 expression in comparison with the lower 30% of patients with low hsa-miR-637 expression **(H)**.

Supplementary Figure 2 The expression of **(A)** *miR-196a-5p*, **(B)** *miR-637*, **(C)** *CYBRD1* and **(D)** *RUFY2* in GBMs and normal tissues in the TCGA database. ^***^*P* < 0.001 vs. control sample.

Supplementary Figure 3 **(A)** PPI network generated using the REACTOME database. **(B)** The PPI network consists of top 300 DEGs in GSE4290.

Supplementary Figure 4 Relationship between the expression of *CYBRD1*/*RUFY2* and *MGMT*/*IDH* status within TCGA GBM samples. The expression of *CYBRD1* in **(A)** methylated/unmethylated *MGMT* and **(B)** wild-type/mutant *IDH* tumors. The expression of *RUFY2* in **(C)** methylated/unmethylated MGMT and **(D)** wild-type/mutant *IDH* tumors.
