# Supplementary material for: LncRNAs Predicted to Interfere With the Gene Regulation Activity of miR-637 and miR-196a-5p in GBM
Source: Front Oncol. 2020 Mar 9;10:303. doi: 10.3389/fonc.2020.00303 (PMC7075452; doi:10.3389/fonc.2020.00303)
Supplement: Supplementary file 1 [file Data_Sheet_1.docx]

# Version info: R 3.2.3, Biobase 2.30.0, GEOquery 2.40.0, limma 3.26.8

# R scripts generated Sat Feb 1 20:01:58 EST 2020

Server: www.ncbi.nlm.nih.gov

Query: acc=GSE25631&platform=GPL8179&type=txt&groups=&colors=&selection=XXXXXXXXXXXXXXXXXXXXXXXXXXXXXXXXXXXXXXXXXXXXXXXXXXXXXXXXXXXXXXXXXXXXXXXXXXXXXXXXXXXXXXX&padj=fdr&logtransform=auto&columns=ID&columns=adj.P.Val&columns=P.Value&columns=F&columns=SEQUENCE&columns=miRNA_ID&columns=SPOT_ID&num=250&annot=submitter

# Unable to generate script analyzing differential expression.

# Invalid input: at least two groups of samples should be selected.

################################################################

# Boxplot for selected GEO samples

library(Biobase)

library(GEOquery)

# load series and platform data from GEO

gset <- getGEO("GSE25631", GSEMatrix =TRUE, getGPL=FALSE)

if (length(gset) > 1) idx <- grep("GPL8179", attr(gset, "names")) else idx <- 1

gset <- gset[[idx]]

# set parameters and draw the plot

dev.new(width=4+dim(gset)[[2]]/5, height=6)

par(mar=c(2+round(max(nchar(sampleNames(gset)))/2),4,2,1))

title <- paste ("GSE25631", '/', annotation(gset), " selected samples", sep ='')

boxplot(exprs(gset), boxwex=0.7, notch=T, main=title, outline=FALSE, las=2)
