# Supplementary figures and images for: LncRNAs Predicted to Interfere With the Gene Regulation Activity of miR-637 and miR-196a-5p in GBM
Source: Front Oncol. 2020 Mar 9;10:303. doi: 10.3389/fonc.2020.00303 (PMC7075452; doi:10.3389/fonc.2020.00303)

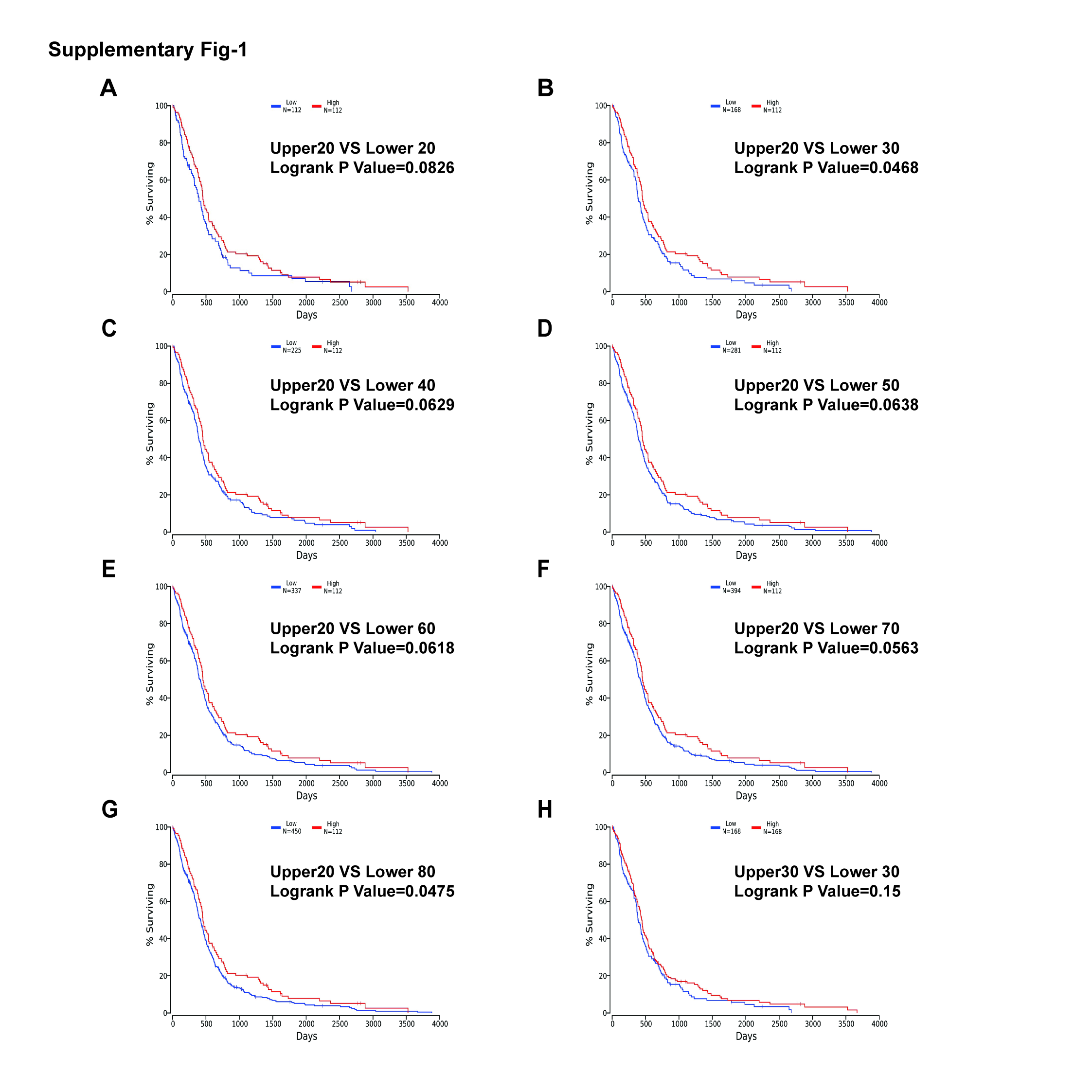

Supplement: Supplementary Figure 1 — Survival curves for the upper 20% of patients with high hsa-miR-637 expression in comparison with the lower 20%, 30%, 40%, 50%, 60%, 70%, and 80% of patients with low hsa-miR-637 expression (A–G). Survival curve for the upper 30% of patients with high hsa-miR-637 expression in comparison with the lower 30% of patients with low hsa-miR-637 expression (H). [file Image_1.TIF]

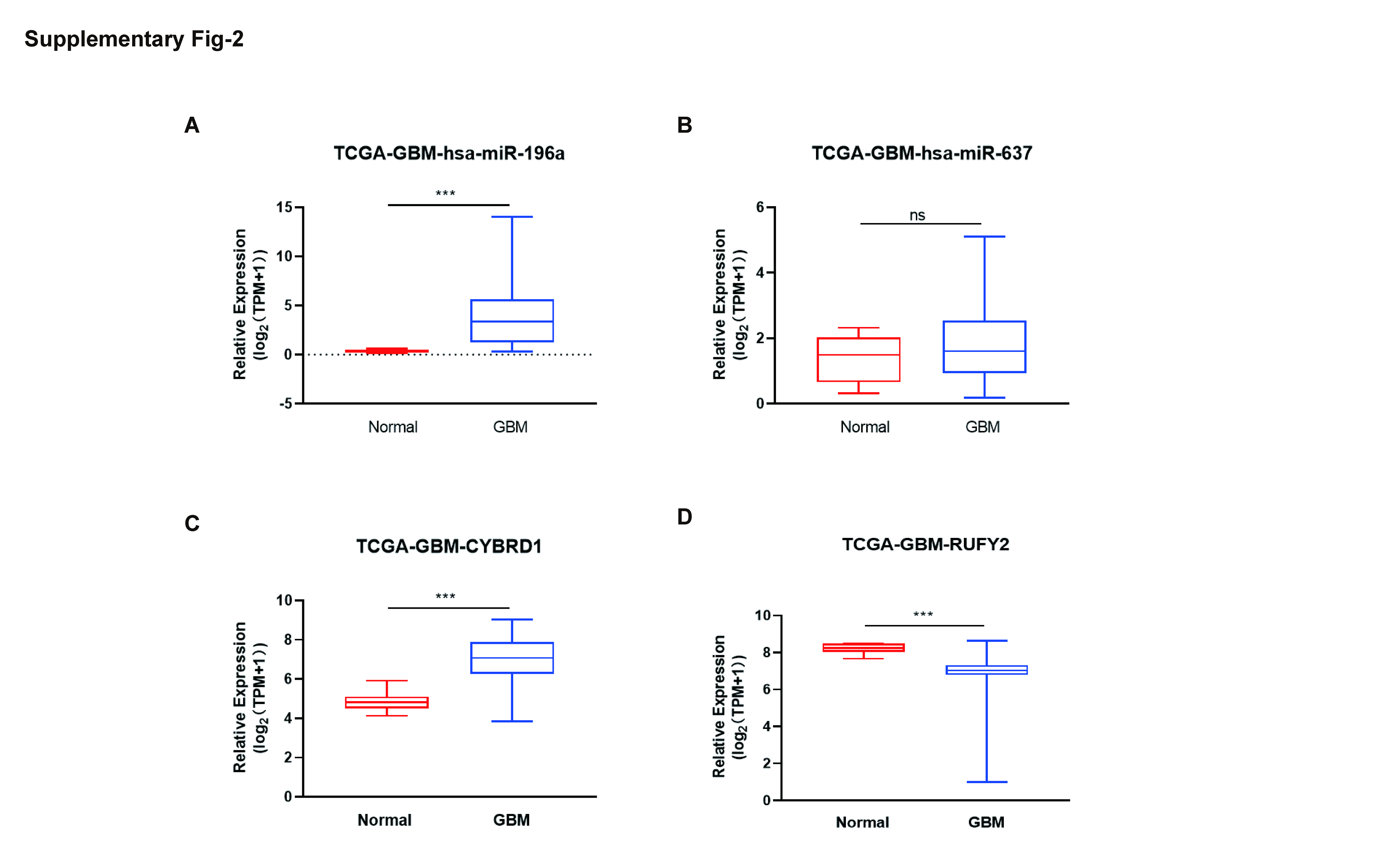

Supplement: Supplementary Figure 2 — The expression of (A) miR-196a-5p, (B) miR-637, (C) CYBRD1 and (D) RUFY2 in GBMs and normal tissues in the TCGA database. ***P < 0.001 vs. control sample. [file Image_2.TIF]

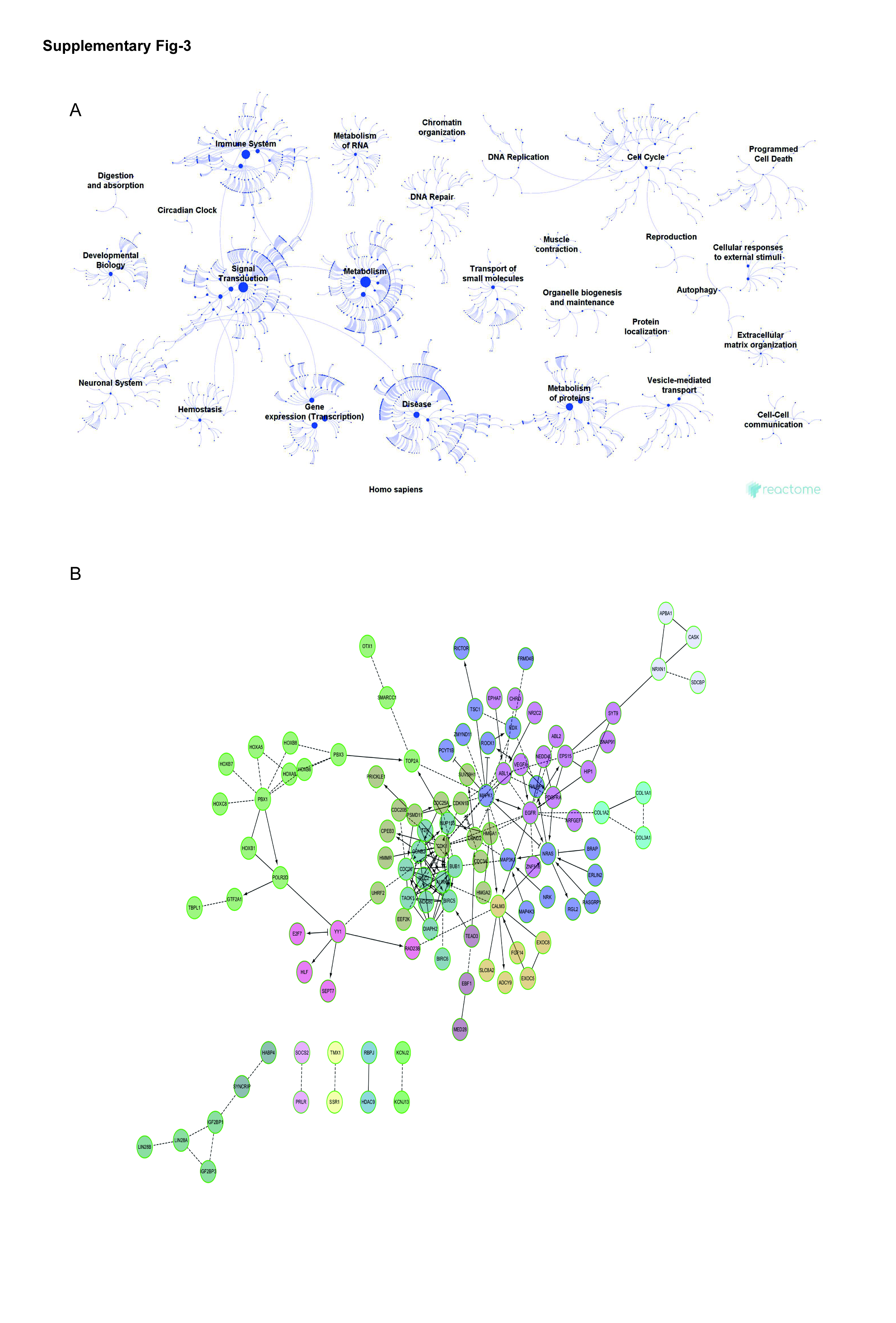

Supplement: Supplementary Figure 3 — (A) PPI network generated using the REACTOME database. (B) The PPI network consists of top 300 DEGs in GSE4290. [file Image_3.TIF]

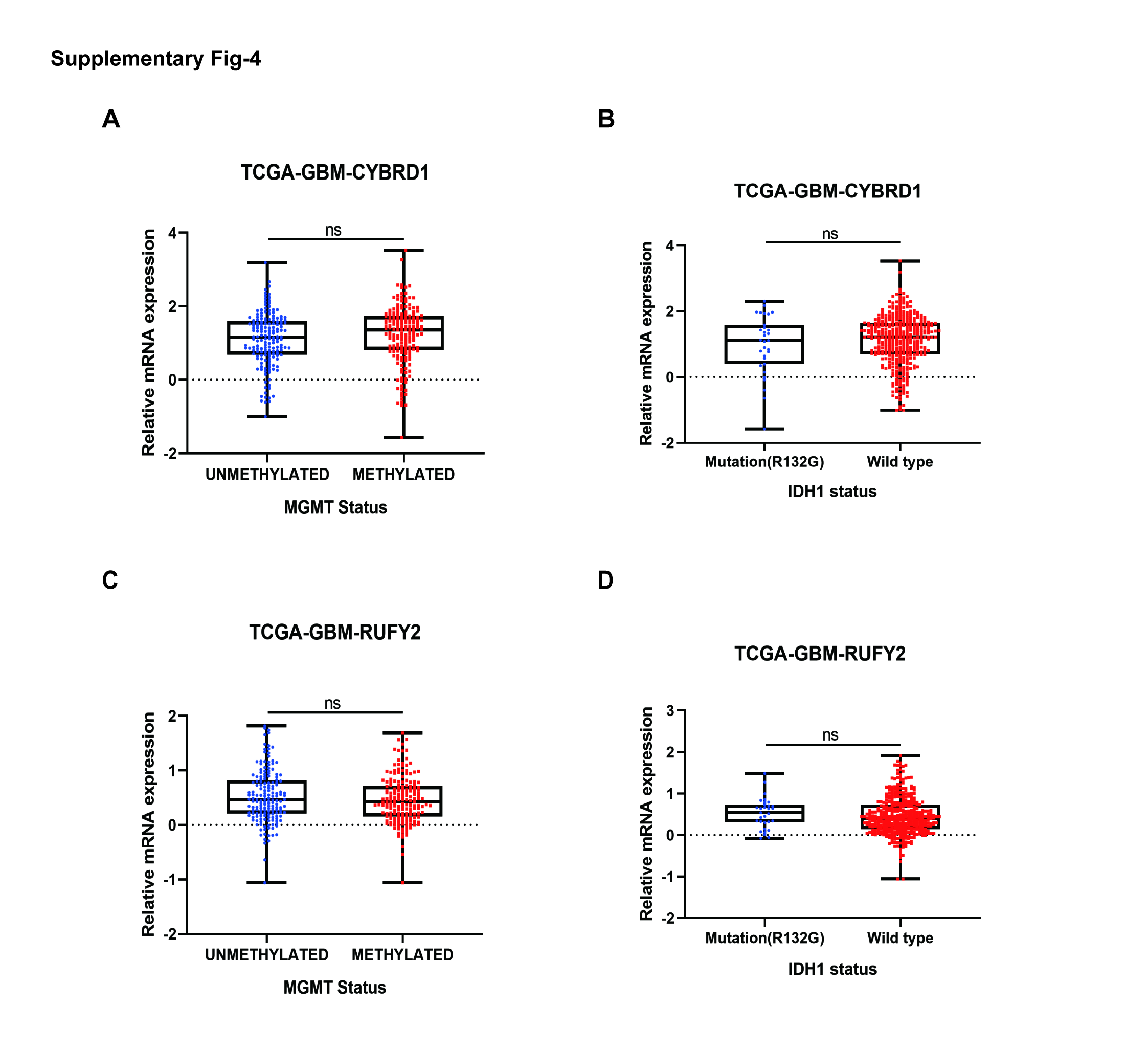

Supplement: Supplementary Figure 4 — Relationship between the expression of CYBRD1/RUFY2 and MGMT/IDH status within TCGA GBM samples. The expression of CYBRD1 in (A) methylated/unmethylated MGMT and (B) wild-type/mutant IDH tumors. The expression of RUFY2 in (C) methylated/unmethylated MGMT and (D) wild-type/mutant IDH tumors. [file Image_4.TIF]
